# Infection characteristics of porcine circovirus type 2 in different herds from intensive farms in China, 2022

**DOI:** 10.3389/fvets.2023.1187753

**Published:** 2023-06-15

**Authors:** Mingyu Fan, Lujie Bian, Xiaogang Tian, Zhiqiang Hu, Weisheng Wu, Leilei Sun, Guiqiang Yuan, Shuangxi Li, Lei Yue, Ying Wang, Lili Wu, Yongquan Wang, Zheng Yan, Jing Ren, Xiaowen Li

**Affiliations:** ^1^Shandong New Hope Liuhe Agriculture and Animal Husbandry Technology Co., Ltd. (NHLH Academy of Swine Research), Dezhou, China; ^2^Shandong Swine Health Data and Intelligent Monitoring Project Laboratory, Dezhou University, Dezhou, China; ^3^Xiajin New Hope Liuhe Agriculture and Animal Husbandry Co., Ltd., Dezhou, China; ^4^New Hope Liuhe Co., Ltd., Chengdu, China

**Keywords:** PCV2, pre-weaning piglet, nursery pig, growing–finishing pig, gilt, sow, positivity rate, viral load

## Abstract

**Introduction:**

Porcine circovirus type 2 (PCV2) is the primary etiological agent of porcine circovirus diseases (PCVD), which are widespread in most pig herds, causing huge economic losses in the global pig industry. Therefore, it is critical to assess the infection characteristics of PCV2 in different swine herds to develop effective strategies against PCVD.

**Methods:**

In this study, routine diagnostic and monitoring protocols were used to collect 12,714 samples from intensive farms in China, and PCV2 was tested for by qPCR to determine positivity rates and viral loads in samples from different herds and materials.

**Results:**

PCV2 was found to be prevalent throughout China, and fattening farms had higher positivity rates than breeding farms. The PCV2 positivity rates in breeding farms in Southern China were higher than those in Northern China. Growing–finishing pigs demonstrated the highest positivity rate in the tested samples, while pre-weaning piglets and adult sows had the lowest. Meanwhile, samples with viral loads exceeding 106 copies/mL in growing–finishing pigs had 27.2% positivity, compared to 1.9% and 3.3% in sows and piglets, respectively. The results of the viral loads in the serum samples followed a similar trend.

**Discussion:**

The findings reveal that PCV2 circulates in different herds from intensive farms, with positivity increasing from pre-weaning to growing–finishing herds. It is urgent to develop effective strategies to reduce PCV2 positivity in growing–finishing herds and prevent viral circulation among pigs.

## Introduction

Porcine circovirus type 2 (PCV2) is a small and ubiquitous single-stranded DNA virus in the genus *Circovirus* of the family *Circoviridae* (1). It is the primary causative agent of porcine circovirus diseases (PCVD), which include subclinical (PCV2-SI), systemic (PCV2-SD), and reproductive (PCV-2-RD) diseases, in addition to porcine dermatitis and nephropathy syndrome (PDNS), resulting in significant economic losses to the swine industry (2). While commercial vaccines have been available for many years, mass vaccination of PCV2 has failed to eradicate the virus (3–5). PCV2 can be detected in the blood, tissue, colostrum, semen, saliva, nasal, fecal, and urinary secretions of pigs, as well as in the environment (6, 7). As a result, PCV2 virus is shed through several different routes and spread between pigs both horizontally and vertically (6, 8, 9), promoting its transmission between different farms and pig herds. Therefore, comprehending the prevalence and dynamics of PCV2 infection is crucial for the efficacious prevention and control of its transmission in diverse pig herds.

A systematic review and meta-analysis study conducted in China revealed that the pooled prevalence of PCV2 was 46.0–50.1% in intensive farms and 37.5% in extensive farms during 2015–2019 (10). However, there is a lack of comprehensive information on the prevalence of PCV2 in various herds and sample types. The current study collected several clinical samples from intensive pig farms across China and analyzed the prevalence of PCV2 infection in different pig farms and herds. The findings help to inform the development of future strategies to prevent the spread of this disease.

## Materials and methods

### Study farms

A total of 131 breeding farms and 91 fattening farms with a two-site production system were selected for this study in China in 2022. The breeding farms, which had a herd composition consisting of pre-weaning piglets (0–21 days of age), gilts (90–230 days of age), adult sows (>230 days of age and in gestation or with a history of gestation), and boars (>300 days of age), had a breeding stock ranging from 750 to 3,000 sows. On the other hand, the fattening farms had a production scale of over 6,000 pigs and included nursery piglets (21–70 days of age) and growing–finishing pigs (70–180 days of age). These farms had similar PCV2 vaccination protocols that were reliably followed. At 14 days of age, the pre-weaning piglets were vaccinated with one dose of the inactivated PCV2 vaccine. The gilts and boars were vaccinated twice with an inactivated vaccine at 14 and 90 days of age. Other age-stage herds did not receive the PCV2 vaccine.

### Sample collection

Samples were collected through routine diagnostic and monitoring procedures across various herds, as shown in Table 1. Umbilical cord blood was obtained from neonatal piglets. Following the delivery of the sows, umbilical cord blood was extracted from their piglets using a syringe and subsequently plastic tube. The collection of testicular processing fluid samples was collected during piglet castration at 5 days of age, whereby approximately 20 liters of piglet testicles were gathered in a plastic bag, and the resulting liquid was transferred into a plastic tube. To obtain oropharyngeal swab samples, a long swab was inserted into the throat and moved back and forth twice. The head of the retrieved swab was then broken off and eluted into a sealed bag with 2 mL of normal saline, and the eluent was transferred into a plastic tube. Placenta plastic tube. Placenta samples were obtained by extracting approximately 5 g of placenta tissue from aborted sows and storing it in a sealed bag. Oral fluid samples were collected by suspending a piece of cotton rope in each pen for the pigs to chew on. After 20 min, the ropes were retrieved and placed in separate plastic bags, and the liquid from each piece was squeezed into a plastic tube. To obtain lymph node samples, the inguinal lymph node tissue of deceased pigs was excised using a scalpel, and 2 g of tissue was taken and placed in a hermetically sealed plastic bag. To obtain serum samples, blood was extracted from the anterior vena cava, left to rest at room temperature for 30 min, and then centrifuged at 1,000 g for 2 min. The serum was subsequently collected in plastic tubes. In pre-weaning piglet herds, oropharyngeal swabs and sera were collected at 20 days of age. Semen samples were exclusively collected from boars. All samples were stored at −20°C.

**Table 1:** Routine diagnostic or monitoring protocols used in pig farms.

**Herd**	**Clinical signs**	**Materials**	**Minimum number sampled**	**Testing frequency**
Pre-weaning piglet	Healthy pigs	Umbilical cord blood	10 pigs	Monthly or by batch
		Testicular processing fluid	Majority of litters from one batch of farrowing	Weekly or by batch
	Weak piglets	Serum	15 pigs	Weekly or by batch
	Weak piglets	Oropharyngeal swabs	15 pigs	Weekly or by batch
Nursery pig	Healthy pigs	Serum	30 pigs	By batch herd test
		Oral fluid	15 pens	By batch herd test
	Dead pigs	Lymph node	3–5 pigs	-
Growing–finishing pig	Healthy pigs	Serum	30 pigs	By batch herd test
		Oral fluid	15 pens	By batch herd test
	Dead pigs	Lymph node	3–5 pigs	-
Gilt	Healthy pigs	Serum	30 pigs	By batch herd test
		Oral fluid	15 pens	By batch herd test
Adult sow	Abortion sows	Placenta	3 pigs	Weekly
	Inactive or poor appetite sows	Oropharyngeal swabs	15 pigs	Weekly
		Serum	30 pigs	Monthly
Boar	Healthy pigs	Semen	All pigs	Monthly

### qPCR

The lymph node or placental tissue (0.5 g) was weighed, 1.5 mL lysis solution was added, and the tissue samples were prepared using Precellys lysing kits with the Precellys tissue homogenizer (Bertin, France). Serum and other liquid samples were oscillated and centrifuged at 5,000 g for 1 min. Total DNA was extracted from 200 μL of each sample using the Virus DNA Extraction Kit II (Geneaid, Taiwan) in accordance with the manufacturer's instructions. Extracted DNA (2.0 μL) was then amplified using real-time PCR of an ORF2 section of PCV2 as previously described (11, 12). Samples with Ct values of < 40 were considered positive. The quantification of viral genome copies was performed using titrated plasmids containing the ORF2 of PCV2 (13). Viral titers inferred from the real-time PCR results were expressed as the viral copy number per milliliter of both the liquid and tissue samples (copies/mL).

### Statistical analysis

Statistical analyses were performed using SPSS Statistics version 22. The positivity rates were represented as absolute and relative frequencies (%) with a 95% confidence interval. The PCV2 DNA copies of each serum sample were analyzed using a one-way ANOVA, with a *P*-value < 0.05 being considered significant.

## Results

### Detection rates of PCV2 in pig farms throughout China

A total of 12,714 clinical samples were collected from diverse pig farms in 18 provinces of China. Among these, 5,075 samples, comprising umbilical cord blood, testicular processing fluid, serum, and oropharyngeal swab samples, were obtained from 131 breeding farms, while 3,877 samples, including oral fluid, lymph node, and serum samples, were collected from 91 fattening farms. Based on the geographical distribution, the 18 provinces were divided into two regions, namely Northern China (Liaoning, Shandong, Tianjin, Hebei, Henan, and Gansu) and Southern China (Jiangsu, Zhejiang, Anhui, Jiangxi, Hubei, Hunan, Sichuan, Chongqing, Guizhou, Guangdong, Guangxi, and Hainan). The positivity rate for PCV2 at the farm level was 46.6% (95% CI: 37.9–55.2%) and 67.0% (95% CI: 57.2–76.9%) in breeding and fattening farms, respectively. In breeding farms, the PCV2 positivity rate was 34.0% (95% CI: 20.0–48.1%) in Northern China and 53.6% (95% CI: 42.7–64.5%) in Southern China. In the fattening farms, the PCV2 positivity rate was 73.9% (95% CI: 60.7–87.1%) in Northern China and 60.0% (95% CI: 45.1–74.9%) in Southern China. At the sample level, the positivity rate was 11.8% (95% CI: 10.9–12.7%) and 40.6% (95% CI: 39.1–42.1%) in the breeding and fattening farms, respectively. In the breeding farms, 3.7% (95% CI: 2.8–4.5%) of the samples in Northern China were PCV2 positive, while 16.1% (95% CI: 14.9–17.4%) in Southern China were positive. In the fattening farms, 42.2% (95% CI: 40.4–44.1%) of the samples were PCV2 positive in Northern China, while 36.7% (95% CI: 34.5–40.1%) were positive in Southern China (Table 2). A total of 3,762 semen samples from boars were collected from 18 of the 131 breeding farms, with only one farm testing positive at a proportion of 0.7% (95% CI: 0.4–1.0%) (Table 3). These findings suggest a potential regional variation in the PCV2 positivity rate in China and a very low positivity rate in boars.

**Table 2:** PCV2 positivity rate in different pig farms throughout China.

**Geographical region**	**Breeding farm**						**Fattening farm**					
	**Tested sample**	**Positive sample**	**Positivity rate at the sample level**	**Tested farm**	**Positive farm**	**Positivity rate at the farm level**	**Tested sample**	**Positive sample**	**Positivity rate at the sample level**	**Tested farm**	**Positive farm**	**Positivity rate at the farm level**
Northern China	1,752	64	3.7% CI: 2.8–4.5%	47	16	34.0% CI: 20.0–48.1%	2,739	1,156	42.2% CI: 40.4–44.1%	46	34	73.9% CI: 60.7–87.1%
Southern China	3,323	536	16.1% CI: 14.9–17.4%	84	45	53.6% CI: 42.7–64.5%	1,138	418	36.7% CI: 34.5–40.1%	45	27	60.0% CI: 45.1–74.9%
Total	5,075	600	11.8% CI: 10.9–12.7%	131	61	46.6% CI: 37.9–55.2%	3,877	1,574	40.6% CI: 39.1–42.1%	91	61	67.0% CI: 57.2–76.9%

Northern China included Liaoning, Shandong, Tianjin, Hebei, Henan, and Gansu; Southern China included Jiangsu, Zhejiang, Anhui, Jiangxi, Hubei, Hunan, Sichuan, Chongqing, Guizhou, Guangdong, Guangxi, and Hainan. A total of 5,075 clinical samples, including umbilical cord blood, testicular processing fluid, serum, and oropharyngeal swab samples, were collected from breeding farms. A total of 3,877 clinical samples, including oral fluid, lymph node, and serum samples, were collected from fattening farms. CI, 95% confidence interval for the positivity rate.

**Table 3:** PCV2 positivity in different pig herds.

**Materials**	**Sample positivity rate % (** ***n*** **) in pig herds**					
	**Pre-weaning piglet**	**Nursery pig**	**Growing–finishing pig**	**Gilt**	**Adult sow**	**Boar**
Serum	1.2% (CI: 0.2–3.0%) (5/404)	13.1% (CI: 9.9–16.3%) (56/429)	37.4% (CI: 34.2–40.5%) (338/904)	44.5% (CI: 40.0–49.0%) (207/465)	3.7% (CI: 1.9–5.6%) (15/403)	/
Oral fluid	/	41.6% (CI: 37.9–45.5%) (270/648)	51.8% (CI: 49.0–54.6%) (631/1,218)	38.6% (CI: 32.3–44.8%) (91/236)	/	/
Placenta of aborted sow	/	/	/	/	1.8% (CI:−0.2–3.8%) (3/168)	/
Oropharyngeal swabs	9.0% (CI: 7.2–10.8%) (85/944)	/	/	/	5.5% (CI: 3.8–7.3%) (36/654)	/
Umbilical cord blood	3.0% (CI: 2.1–3.9%) (40/1,342)	/	/	/	/	/
Testicular processing fluid	25.7% (CI: 21.7–29.7%) (118/459)	/	/	/	/	/
Lymph node	/	39.4% (CI: 33.9–44.9%) (121/307)	42.6% (CI: 37.5–47.6%) (158/371)	/	/	/
Semen	/	/	/	/	/	0.7% (0.4–1.0%) (26/3,762)
Total	7.9% (CI: 6.9–8.8%) (248/3,149)	32.3% (CI: 29.8–34.8%) (447/1,384)	45.2% (CI: 43.3–47.2%) (1,127/2,493)	42.5% (CI: 38.8–46.2%) (298/701)	4.4% (CI: 3.3–5.6%) (54/1,225)	0.7% (CI: 0.4–1.0%) (26/3,762)

CI, 95% confidence interval for the sample positivity rate.

### PCV2 positivity in different pig herds by sample type

Samples were obtained from diverse pig herds encompassing all ages and genders of pigs (Table 3). The highest rates of detection were observed in growing–finishing pigs (45.2%, 95% CI: 43.3–47.2%), followed by gilt sows (42.5%, 95% CI: 38.8–46.2%) and nursery pigs (32.3%, 95% CI: 29.8–34.8%). Conversely, pre-weaning piglets (7.9%, 95% CI: 6.9–8.8%), adult sows (4.4%, 95% CI: 3.3–5.6%), and boars (0.7%, 95% CI: 0.4–1.0%) exhibited relatively low rates of detection. Regarding serum samples from the different pig herds, PCV2 positivity was highest in gilt sows (44.5%, 95% CI: 40.0–49.0%), followed by growing–finishing pigs (37.4%, 95% CI: 34.2–40.5%). Nursery pigs, adult sows, and pre-weaning piglets displayed positivity rates of 13.1% (95% CI: 9.9–16.3%), 3.7% (95% CI: 1.9–5.6%), and 1.2% (95% CI: 0.2–3.0%), respectively. The testicular processing fluid sample had a positivity rate of 25.7% (95% CI: 21.7–29.7%), indicating that these samples were the most reliable for monitoring PCV2 infection in pre-weaning piglets. In nursery pigs, oral fluid samples had the highest positivity rates (41.6%, 95% CI: 37.9–45.5%), followed by lymph nodes (39.4%, 95%CI: 33.9–44.9%). Among growing–finishing pigs, both oral fluid and lymph node samples had high positivity rates of 51.8% (95% CI: 49.0–54.6%) and 42.6% (95% CI: 37.5–47.6%), respectively, indicating a high rate of infection in these herds. In addition, all the samples in the adult sows had low positivity rates, and the positivity rate in the placenta samples was only 1.8% (95% CI: −0.2–3.8%). Boars were only sampled for semen, which exhibited a very low positivity rate of 0.7% (95% CI: 0.4–1.0%).

### PCV2 viral loads in different pig herds

**Figure 1:**
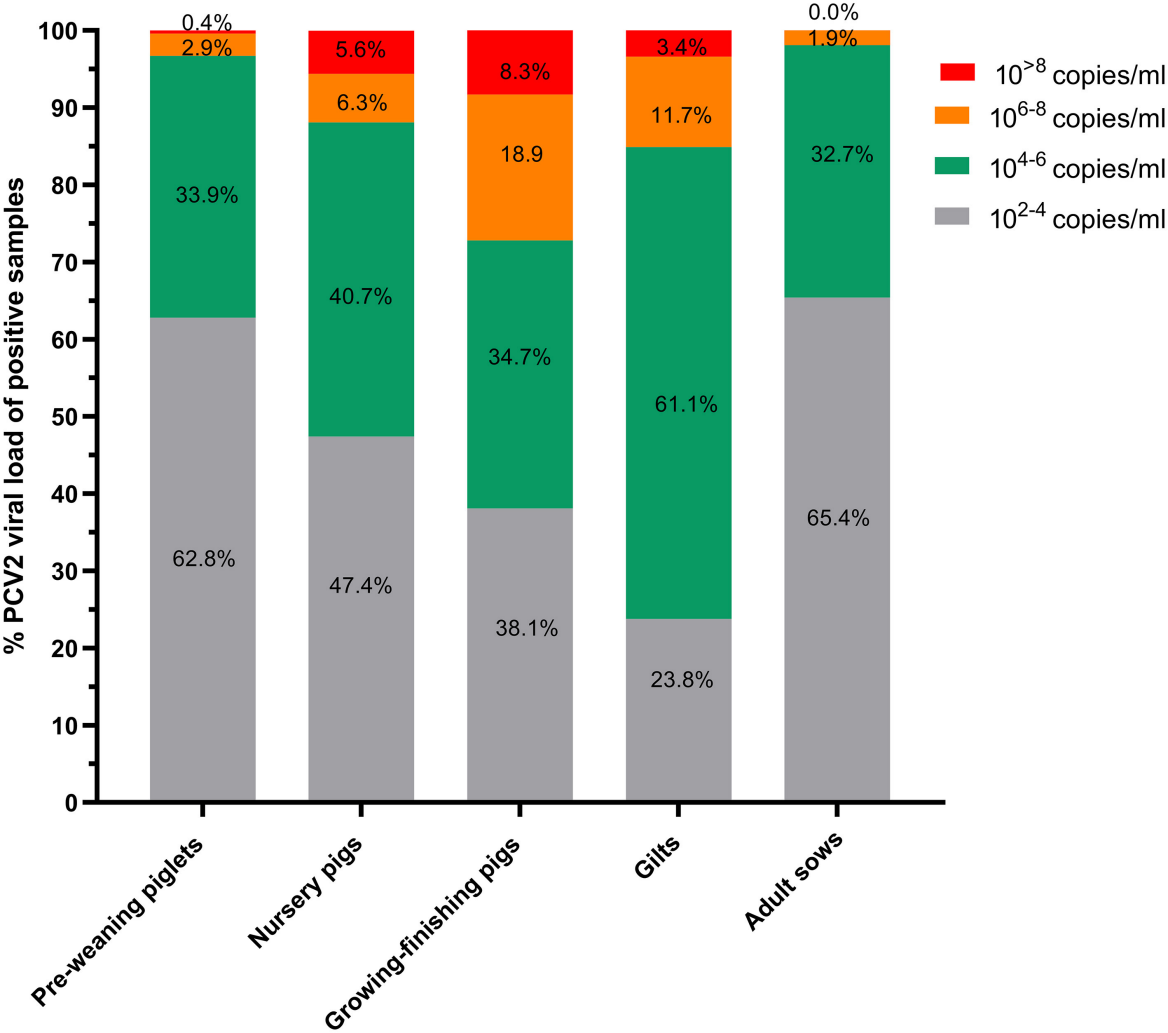
The percentage of PCV2-positive samples by PCV2 copy number in different herds. PCV2 DNA was isolated from all PCV2-positive samples, and the viral copy number was determined by qPCR using a standard curve. The results are expressed as the viral copy number per milliliter of liquid and tissue sample (copies/mL).

Viral loads were divided into four gradients, containing 10^2 − 4^, 10^4 − 6^, 10^6 − 8^, and 10^>8^ viral particles, and the proportion of samples within each gradient was calculated. More than 90% of the positive samples in the adult sows and pre-weaning piglets had viral loads < 10^6^, and most were in the range of 10^2 − 4^ (Figure 1). More samples in the 10^4 − 6^ gradient were found in nursery pigs, growing–finishing pigs, and gilts than in adult sows and pre-weaning piglets. Notably, >15% of the positive samples in the growing–finishing pigs and gilts exhibited viral loads >10^6^, with some samples exhibiting viral loads >10^8^. Specifically, 8.3% of the samples from the growing–finishing pig herds exhibited viral loads >10^8^, followed by 5.6% of samples from the nursery pig herds.

**Figure 2:**
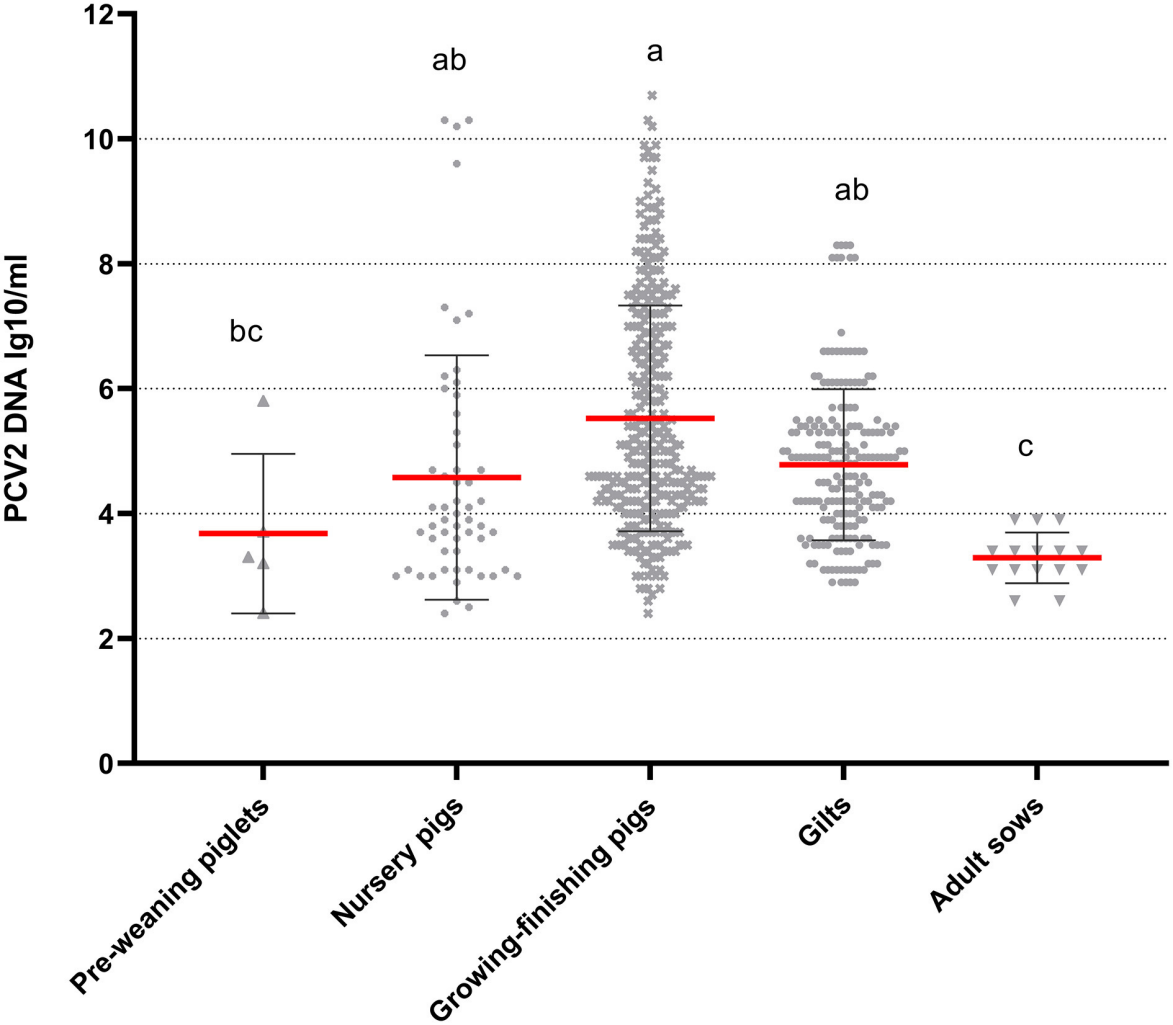
Viral loads of PCV2-positive serum samples in different herds. The results are expressed as the log10 viral copy number per milliliter of serum sample (copies/mL). Mean values with superscript letters a, b, and c are with significant differences across groups. Different letters indicate significant statistical differences (*P* < 0.05), and the same letter indicates no significant statistical differences (*P* > 0.05).

The analysis of the serum samples collected from the various herds was conducted independently. Viral loads were highest in the samples from the growing–finishing pigs but did not significantly differ from those of the nursery pig and gilt samples (*P* > 0.05) (Figure 2). The pre-weaning piglet herds, nursery herds, and gilt herds did not exhibit significant difference (*P* > 0.05), while adult sow herds had the lowest viral loads (*P* < 0.05). The observed trends in viral loads were consistent with the PCV2 positivity rates (Table 3).

## Discussion

The expansion of intensive pig farms and the migration of diverse pig herds across China in recent years have necessitated the development of more effective prevention and control strategies for PCV2. This study found that PCV2 has spread widely in intensive pig farms, with a higher positivity rate observed in fattening farms, corroborating the findings of Liu et al. (10) and Li et al. (14). However, the positivity rates of PCV2 in breeding and fattening farms differ between Northern and Southern China (Table 2). Southern China has a higher positivity rate of PCV2 in breeding farms. This phenomenon may be attributed to differences in the geographical environment, climate, and protocols implemented since 2018 to contain African swine fever (15–17); these factors can impact the construction density, pig movement management, environmental control strategies, biosafety level, and disease prevention procedures of breeding and fattening farms.

Semen transmission is considered an important route of PCV2 transmission (6, 18, 19). However, the present study revealed that only 0.7% of the 37,62 semen samples collected were found to be positive for PCV2, and the viral loads of the positive samples were observed to be very low (Table 3). These findings suggest that semen transmission may not be a primary route for the spread of PCV2 in intensive pig farms. Unfortunately, other sample types were not collected from the boars in this study, so additional information regarding PCV2 infection in boars is not available.

The detection rate of the PCV2 genome in serum samples from pre-weaning piglets was low at 1.2%, while the detection rate in testicular processing fluid samples was significantly higher at 25.7%. These findings align with previous research by Dieste-Pérez et al. (20), and they suggest that testicular processing fluids may be a more effective means of monitoring PCV2. In addition, the high positivity of testicular fluids indicates that infection occurs early in piglets, and the risk of PCV2-associated diseases is high after weaning (21). The results of this study also indicate a higher proportion of PCV2-positive samples in nursery and growing–finishing pigs compared to pre-weaning piglets (Table 3), which suggests that PCV2 carried by weaned piglets was being transferred from breeding to finishing farms and transmitted horizontally through continuous contact between pigs (22). The co-mingling of pigs of different ages and the exchange of animals, people, equipment, and sundries between barns or herds may contribute to the horizontal transmission of PCV2 in such intensive farming environments (6, 23). At the same time, the gilt samples had a higher PCV2 positivity rate, suggesting that gilts from the quarantine and rearing areas played a major role in the spread of PCV2 and the maintenance of infection in sow herds (24).

The serum and lymph node samples collected from the nursery and growing–finishing pigs were analyzed for positivity rates of PCV2. The results indicate that the lymph node samples had a higher positivity rate (39.4%) compared to the serum samples (13.1%) in the nursery, which may be attributed to the virus's preferential targeting of immune cells in the lymphoid tissue without causing extensive viremia (25). Furthermore, the lymph node samples from the growing–finishing pigs had a higher positivity rate (42.6%), indicating that PCV2-associated immunosuppressive diseases affected pig growth during the nursery and growing–finishing periods. These findings also suggest that oral fluid samples are good indicators of PCV2 infection in nursery and growing–finishing pigs and could serve as reliable material for PCV2 detection at the environmental and individual pig levels (26).

The main difference between subclinical and clinical PCVD is the severity of the lesion, which correlates with the viral load in samples, especially in the sera and tissues (27, 28). Viral loads >10^6^ copies/mL are believed to strongly influence the development of PCVD (12). In adult sow herds and pre-weaning piglet herds, only 1.9% and 3.3% of PCV2-positive samples, respectively, exhibited viral loads >10^6^, indicating that PCVD was mild during the farrowing period. There was a significant increase in samples with viral loads >10^6^ copies/mL in both the growing–finishing pig and gilt herds, with 8.3% of growing–finishing herds having viral loads >10^8^ copies/mL, suggesting that PCVD was severe in the two herds. In addition, fewer samples from the gilts than from the growing–finishing pig herds had viral loads >10^6^ copies/mL, which may be because the gilts had received two PCV2 vaccine doses at 14 and 90 days of age.

A serum viral load has been established as a reliable indicator of both PCV2-associated diseases and average daily weight gain (ADWG) (27, 29, 30). The present study observed a gradient of PCV2 in serum samples from various herds, as depicted in Figure 2. This trend was consistent with the PCV2 positivity rates reported in Table 3, which is in line with the findings of López-Soria et al. (29). Furthermore, a standard was also established for viral loads in serum, with samples >10^5.3^ being considered as high (29). The growing–finishing herds in this study exhibited serum samples with high viral loads, necessitating the development and implementation of corrective measures.

In summary, the present study has demonstrated that PCV2 circulates in diverse herds, with its incidence increasing from pre-weaning herds to growing–finishing herds. These findings illustrate that growing–finishing herds have the highest risk of PCVD, indicating the need for effective strategies to reduce the positivity rate of PCV2 in growing–finishing herds and prevent viral circulation among pigs.

## Data availability statement

The original contributions presented in the study are included in the article/supplementary material, further inquiries can be directed to the corresponding authors.

## Author contributions

XL and JR conceived and designed the experiments. XL, JR, MF, LB, and XT supervised and conducted the experiment and analyzed the data. MF, LB, and XT wrote the original draft. XL reviewed and edited the manuscript. ZH, WW, LS, GY, SL, LY, LW, YiW, YoW, and ZY contributed to the samples and methods. All authors have read and approved the final manuscript.
